# Structural and functional analysis of the Rpf2-Rrs1 complex in ribosome biogenesis

**DOI:** 10.1093/nar/gkv305

**Published:** 2015-04-08

**Authors:** Nozomi Asano, Koji Kato, Akiyoshi Nakamura, Keisuke Komoda, Isao Tanaka, Min Yao

**Affiliations:** 1 Graduate School of Life Science, Hokkaido University, Sapporo 060-0810, Japan; 2 Faculty of Advanced Life Science, Hokkaido University, Sapporo 060-0810, Japan

## Abstract

Proteins Rpf2 and Rrs1 are required for 60S ribosomal subunit maturation. These proteins are necessary for the recruitment of three ribosomal components (5S ribosomal RNA [rRNA], RpL5 and RpL11) to the 90S ribosome precursor and subsequent 27SB pre-rRNA processing. Here we present the crystal structure of the *Aspergillus nidulans* (*An*) Rpf2-Rrs1 core complex. The core complex contains the tightly interlocked N-terminal domains of Rpf2 and Rrs1. The Rpf2 N-terminal domain includes a Brix domain characterized by similar N- and C-terminal architecture. The long α-helix of Rrs1 joins the C-terminal half of the Brix domain as if it were part of a single molecule. The conserved proline-rich linker connecting the N- and C-terminal domains of Rrs1 wrap around the side of Rpf2 and anchor the C-terminal domain of Rrs1 to a specific site on Rpf2. In addition, gel shift analysis revealed that the Rpf2-Rrs1 complex binds directly to 5S rRNA. Further analysis of Rpf2-Rrs1 mutants demonstrated that *Saccharomyces cerevisiae* Rpf2 R236 (corresponds to R238 of *An*Rpf2) plays a significant role in this binding. Based on these studies and previous reports, we have proposed a model for ribosomal component recruitment to the 90S ribosome precursor.

## INTRODUCTION

The ribosome is responsible for the translation of messenger RNA (mRNA) into proteins. Eukaryotic ribosomes comprise four different ribosomal RNAs (rRNAs) and approximately 80 ribosomal proteins. The biogenesis of this large nucleoprotein particle is complex and requires more than 200 different proteins and RNA/protein complexes (1–3).

rRNAs are generated from two independent transcription units. RNA polymerase I synthesizes 35S pre-rRNA, a long rRNA precursor that encompasses 18S, 5.8S and 25S rRNA, whereas RNA polymerase III transcribes 5S rRNA. 35S primary transcripts are packaged into a 90S ribonucleoprotein particle (90S precursor) together with many ribosomal proteins and assembly factors. Next, the 35S pre-rRNA is cleaved at internal transcribed spacer 1, located between the 18S and 5.8S rRNA, to generate the 20S and 27SA_2_ pre-rRNA intermediates. These steps determine the splitting of the 90S precursor into two independent complexes, the pre-40S and pre-60S ribosomal particles. The 5′ cleavage of 27SA_2_ generates 27SB, which is further divided into 7S and 25.5S (the precursors of 5.8S and 25S rRNAs, respectively). Subsequently, ribosomal subunits exit the nucleolus through the nucleoplasm to the cytoplasm, where they are further assembled into 80S ribosomes for translation (4).

27SB pre-rRNA processing is a point of control in ribosome biogenesis; nascent ribosomes are released from the nucleolus into the nucleoplasm once this step has been completed (5). This step is thought to require major conformational pre-rRNP rearrangements (6,7). Fourteen assembling factors have been shown to be necessary for 27SB pre-rRNA processing (8). Rpf2 and Rrs1 are two such factors.

Rpf2 is an Imp4 superfamily protein. Although proteins in this family play distinct roles at different stages of ribosome biogenesis, all have a similar domain architecture consisting of a central globular Brix domain and optional highly charged N- and C-terminal segments (9). The Brix domain consists of a short peptide that is highly related to a DNA-binding motif in the *Escherichia coli* σ^70^ transcription factor and is considered to be a eukaryotic RNA-binding domain (10). Brix domain function was characterized in a study of Imp4. The Imp4/Brix domain can associate with U3 small nucleolar RNA (snoRNA) and Mpp10, which are required for 35S pre-rRNA processing, to release 18S pre-rRNA and thus single-handedly initiate small subunit biogenesis (11,12). Moreover, single-stranded telomeric DNA has been reported to bind to the Imp4/Brix domain (13). A structural analysis of the Imp4-like protein Mil revealed the characteristic architecture of the Brix domain, which features a similar arrangement of structural elements in its N- and C-terminal halves (14). Although the Brix domain serves as a scaffold for interactions with several binding partners, these interactions are not well understood.

Rrs1 was isolated as a factor related to a secretory defect that caused the transcriptional repression of both rRNA and ribosomal protein genes (15). This protein was known to localize to the nucleolus and nuclear periphery. In the nucleolus, Rrs1 acts as a ribosome assembly factor. At the nuclear periphery, it directly interacts with the membrane-spanning SUN domain protein Mps3 and silent information factor Sir4, which are involved in telomere clustering and silencing (16).

In 2007, an Rpf2-subcomplex comprising two assembly factors (Rpf2 and Rrs1), two ribosomal proteins (RpL5 and RpL11) and 5S rRNA was isolated from *Saccharomyces cerevisiae* (*Sc*) (17). These factors assembled into a 90S precursor containing 35S pre-rRNA (17). Genetic depletion of each of the four proteins inhibited the recruitment of the other three proteins and 5S rRNA to the 90S precursor (17). Moreover, depletion blocked the conversion of 27SB pre-rRNA to 7S and 25.5S pre-rRNA (10,18). These results suggested that Rpf2 and Rrs1 load 5S rRNA, RpL5 and RpL11 into the 90S precursor. 27SB pre-rRNA processing occurs subsequent to this recruitment, indicating that the correct recruitment of 5S rRNA, RpL5, RpL11, Rpf2 and Rrs1 is a checkpoint of ribosome biogenesis. Yeast two-hybrid assays and GST pull-down experiments revealed direct interactions between these proteins (17–21). Among these interactions, the strong interaction between Rpf2 and Rrs1 suggested that these proteins function as a heterodimer. Although L65 in *Sc*Rrs1 is known to be critical for interactions of Rrs1 with RpL5 and RpL11 (21), details of the other interactions are not clear. Furthermore, it is also unknown whether the Rpf2-Rrs1 complex directly associates with 5S rRNA.

We conducted a structural and functional analysis of the Rpf2-Rrs1 complex. Here we describe the crystal structure of the Rpf2-Rrs1 core complex, which contains the N-terminal domains of both Rpf2 and Rrs1. The complex structure revealed that the N-terminal domains of both Rpf2 and Rrs1 interact tightly at three regions. Interestingly, the highly conserved proline-rich loop of Rrs1 wraps around Rpf2 and facilitates separation of the N- and C-terminal domains of Rrs1. We also demonstrated the direct binding of the Rpf2-Rrs1 complex to 5S rRNA. Moreover, the binding region of Rpf2 necessary for 5S rRNA recognition was identified. These results show that the N-terminal domain of Rpf2 plays a critical role in binding to both 5S rRNA and Rrs1.

## MATERIALS AND METHODS

### Constructs and protein expression and purification

Preparation of the *Aspergillus nidulans* (*An*) Rpf2-Rrs1 complex has been described previously (22). The size exclusion chromatography showed that the stoichiometry of (*An*) Rpf2-Rrs1 complex is 1:1 in solution. The coding sequences for *Sc*Rpf2 were amplified by polymerase chain reaction (PCR) and inserted into pET28a (Novagen/Merck Millipore, Billerica, MA, USA) modified by the addition of D-box fused with an N-terminal His 6 tag (cleavable by TEV protease). The plasmids for *Sc*Rpf2 C-terminal deletion mutant (*Sc*Rpf2ΔC; deletion of 90 C-terminal residues, 255–344) and point mutation variants were constructed by the inverse PCR method. Details of the templates and primers used in this study are described in the Supplementary Tables S1 and S2.

The coding sequences for *Sc*Rrs1 were amplified by PCR and inserted into pCDF Duet1 (Novagen) that was modified by the addition of D-box. The plasmid for the *Sc*Rrs1 C-terminal deletion mutant (*Sc*Rrs1ΔC; deletion of 94 C-terminal residues, 110–293) was constructed using an inverse PCR method with the *Sc*Rrs1 expression vector as a template. All vectors were confirmed by plasmid DNA sequencing.

The expressed *Sc*Rpf2-Rrs1 full-length complex was purified using a previously described method (22). Instead of a Resource S column, we used a HisTrap heparin column (GE Healthcare, Little Chalfont, UK) and performed size-exclusion chromatography with a different SEC buffer (20 mM HEPES-NaOH pH 7.5, 300 mM NaCl, 10 mM MgCl_2_ and 10% (v/v) glycerol). Three C-terminal deletion complexes (*Sc*Rpf2ΔC-Rrs1, *Sc*Rpf2-Rrs1ΔC, *Sc*Rpf2ΔC-Rrs1ΔC) were prepared using the same method.

The point-mutated *Sc*Rpf2 variants and *Sc*Rpf2ΔC were co-expressed with *Sc*Rrs1ΔC. The expressed complexes were purified on a HisTrap HP column and HiLoad 16/60 Superdex 200-pg column, followed by dialysis against dialysis buffer (20 mM HEPES-NaOH pH 7.5, 300 mM NaCl, 10 mM MgCl_2_, 1 mM DTT, 50% (v/v) glycerol); the purified products were stored at −30ºC.

### In vitro rRNA transcription

*Sc*5S rRNA was prepared via *in vitro* transcription using T7 RNA polymerase. The DNA oligonucleotides used to construct *Sc*5S rDNA are described in Supplementary Table S2. The 5S_S1∼6 and 5S_AS1∼6 primers were mixed and annealed after heating at 90ºC for 5 min, followed by a subsequent cool down to 4ºC at 0.1ºC/12 sec. The fragment was cloned into pUC19 via the EcoRI and HindIII sites. The 5S rDNA sequence was confirmed by plasmid DNA sequencing. Double-strand DNA transcription templates were obtained via PCR with 5S_S-1 and 5S_AS-7. In vitro transcription was performed overnight at 37ºC in a solution containing 80 mM HEPES-NaOH pH 8.1, 20 mM MgCl_2_, 2.04 mM spermine, 20 mM DTT, 40 mM KCl, 1.4 μg/ml BSA, 5 mM NTPs, 20 mM GMP, 2.5 μg/ml transcription template, 0.1 U/ml pyrophosphatase and 0.24 mg/ml T7 RNA polymerase. The reaction mixture was subsequently isopropanol-precipitated, purified by denaturing urea-polyacrylamide gel electrophoresis and extracted with an Elutrap Electroelution system (Whatman plc, Maidstone, UK). Pooled 5S rRNAs were precipitated with ethanol and resuspended in RNA buffer (20 mM Tris-HCl pH 7.5, 300 mM NaCl, 10 mM MgCl_2_, 10% (v/v) glycerol).

### Structure determination

Crystallization of and data collection from a proteolytic-resistant complex comprising the N-terminal domains of *An*Rpf2 (18–262) and *An*Rrs1 (10–113) were described previously (22). The structure of the Rpf2-Rrs1 core complex was determined by a single wavelength anomalous diffraction (SAD) method using Se-Met substituted protein crystals at a resolution of 3.5 Å. All 10 selenium sites were identified and used for phasing by Autosol (Phenix) (23). After density modification, the initial model was built using Autobuild (Phenix) (24) and contained a single Rrs1 chain (residues17–92) and some Rpf2 fragments. This model was used for the structural analysis of the native Rpf2-Rrs1 crystal at 2.35 Å resolution by molecular replacement using Automr (Phenix) (25). Several rounds of refinement were performed by alternating Refine (Phenix) (26) with manual fitting and rebuilding based on 2Fo−Fc and Fo−Fc electron density maps constructed using COOT (27). The final *R*_free_ and *R*_work_ were 23.5% and 19.2%, respectively. The final refinement statistics and geometry are summarized in Supplementary Table S3. All structure figures were generated using PyMol (The PyMOL Molecular Graphics System, Version 1.3 Schrödinger, LLC). Figures for sequence conservation and surface potentials were produced using Consurf (28) and APBS (29). Sequence alignments were performed using CLUSTALW (30) and alignment figures were prepared using program ESPrint (31).

### Gel shift assays of Rpf2-Rrs1 mutants with 5S rRNA

Full-length 5S rRNA (50 pmol) in 5 μL of RNA buffer was mixed with increasing amounts of individual Rpf2-Rrs1 mutants (50, 100, 200 pmol) in 5 μL of SEC buffer or dialysis buffer. After pre-incubation at 37ºC for 15 min, each sample was subjected to 5% polyacrylamide (acrylamide/bisacrylamide ratio 39/1) gel electrophoresis. The electrophoresis conditions were as follows: temperature, 4ºC; power voltage, 100 V; and electrophoresis buffer, 192 mM glycine and 25 mM Tris buffer. The gels were stained with ethidium bromide or Coomassie brilliant blue R-250.

### Circular dichroism spectrum

Purified proteins were dialyzed against 10 mM Tris-HCl pH 7.5, 20 mM NaCl and 10% (v/v) glycerol. Circular dichroism (CD) spectra were measured on a J800 spectropolarimeter (Japan Spectroscopic Company) in a quartz cell with an optical path length of 2 mm. The CD spectra were obtained by taking the average of four scans taken in the range of 300–190 nm and normalized to molar ellipticities using the protein concentrations.

## RESULTS

### Overall structure

**Figure 1.**
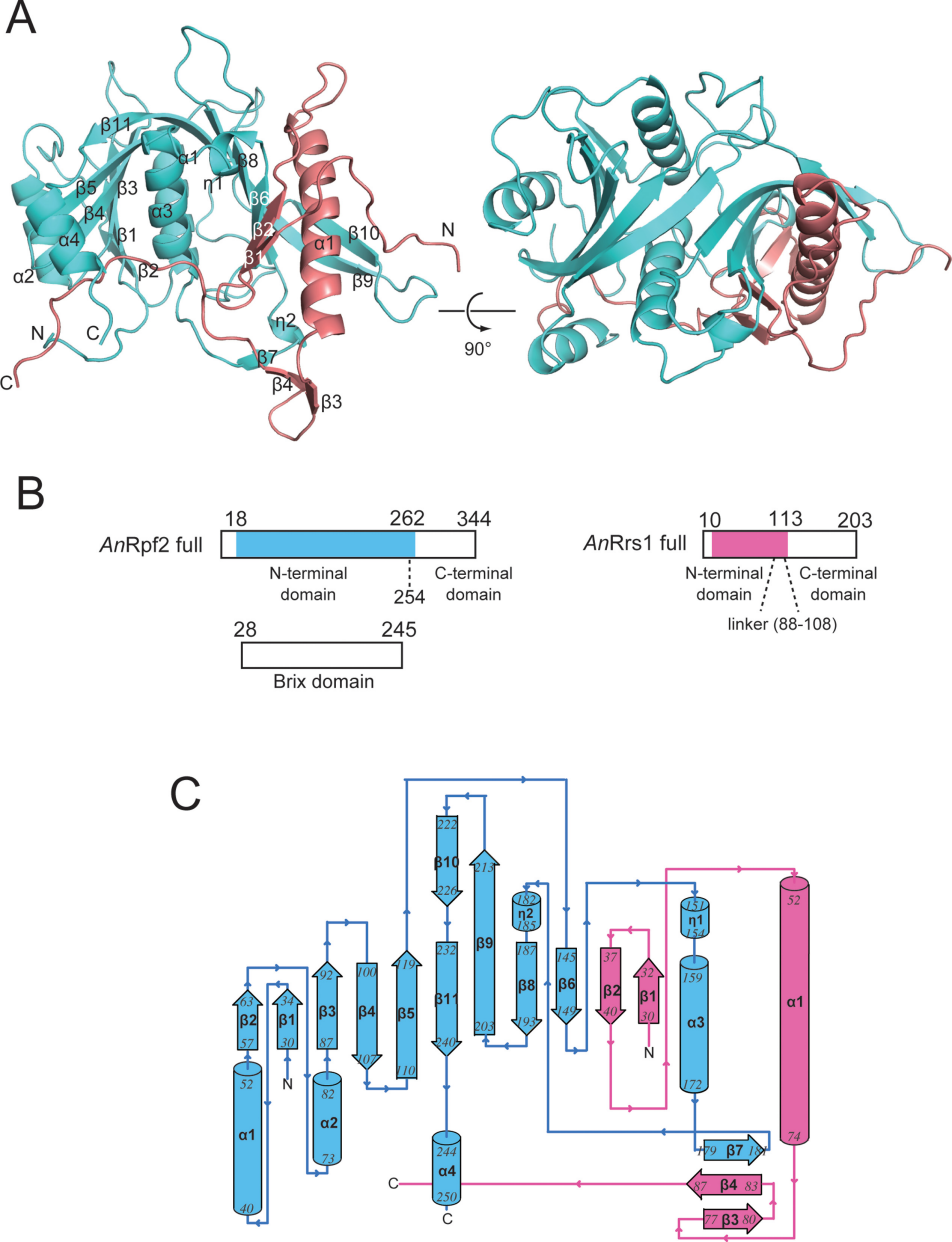
Overall structure of the Rpf2-Rrs1 core complex. Rpf2 and Rrs1 are shown in cyan and pink, respectively. (**A**) Ribbon diagram of the Rpf2-Rrs1 core complex viewed from the front (left) and top (right) of the complex. (**B**) Schematic drawings indicating each domain. The proteolysis resistant complex (Rpf2-Rrs1 core complex) used for this experiment is indicated by color regions. (**C**) Topology diagram indicating the secondary structure. Helices and beta strands are numbered.

A proteolysis-resistant complex of *An*Rpf2 and *An*Rrs1 (Rpf2-Rrs1 core complex), comprising residues 18–262 of *An*Rpf2 and residues 10–113 of *An*Rrs1 (Figure 1B), was crystallized into an orthorhombic *P*2_1_2_1_2_1_ space group with the unit-cell parameters *a* = 54.1, *b* = 123.3, *c* = 133.8 Å (22). The Rpf2-Rrs1 core complex structure was determined to a resolution of 2.35 Å (Figure 1A). The crystal contained two independent Rpf2-Rrs1 core complexes (complex-1: chain A(Rpf2)-B(Rrs1) and complex-2: chain C(Rpf2)-D(Rrs1)). In the crystal, chain B (Rrs1) and chain C (Rpf2) were also connected by β-sheet-type hydrogen bonds (between β3 of Rrs1 and β2 of Rpf2). Similarly, chain A (Rpf2) and chain D (Rrs1) were connected by the same type of hydrogen bonds. However, extensive interactions between chains A and B and chains C and D clearly identify the physiological pair. The structures of these two independent complexes were nearly identical (RMSD for all Cα atoms was 0.23 Å). As the electron density for complex-1 was more clearly visible than that for the complex-2 (Residues 19–254 for Rpf2 and residues 17–104 for Rrs1 were built for complex-1, whereas two fewer N-terminal residues were built for Rrs1 of complex-2), we have described the structure based on complex-1.

Rpf2 exhibited an α + β structure. The structure comprised a wide, twisted half β-barrel in which 10 β-strands were surrounded by four α-helices and two 3_10_ turn helices (Figure 1A and C). The N-terminal domain (residues 19–254) of *An*Rpf2 included the Brix domain (residues 28–245) as predicted by PROSITE (32). A structural similarity search of the DALI server (33) revealed that Rpf2 resembles the Imp4-like protein Mil (PDB ID:1W94 (14); Z score = 9.2), which contains a Brix domain. The rmsd value is 3.20 Å for Cα atoms of 141 residues. However, the Brix domains of Rpf2 and Mil diverged considerably (see discussion). Rrs1 was composed of a single long α-helix (residues 52–74), four short β-strands and a long C-terminal loop (residues 88–104) (Figure 1A and C). The structure was mainly stabilized by interactions with Rpf2. The hydrophobic core was also formed in Rrs1 via intra-molecular interactions of the conserved residues on the C-terminal region of the helix and the residues at the loop region (L33, L36, A38, L67, L71 and L88). The long α-helix was also stabilized via intra-molecular hydrogen bonds between largely conserved side chains (Q65-N69, Q65-R61 and R61-D62).

### Detailed interaction between Rpf2 and Rrs1

**Figure 2.**
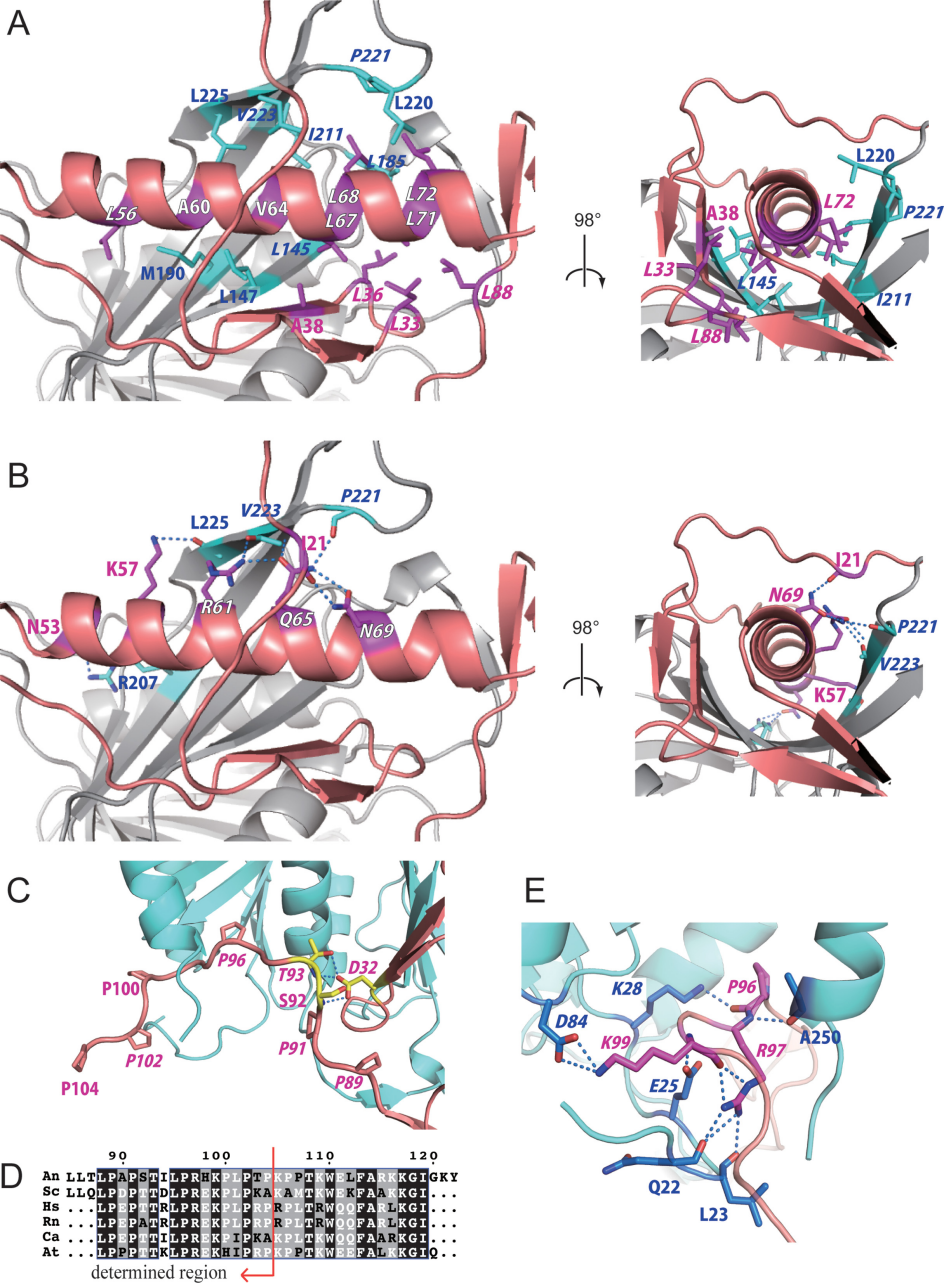
Interactions between Rpf2 and Rrs1. (A and B) Dimerization interface between the long α-helix of Rrs1 and wide β-sheet of Rpf2. Residues of Rrs1 (purple) and Rpf2 (cyan) involved in the interactions are displayed as stick models and labeled. (**A**) Hydrophobic interactions. (**B**) Hydrogen-bonding interactions. (**C**) Close-up view of the C-terminal proline-rich loop of Rrs1. Prolines are labeled and displayed as stick models. Side chains of D32, S92 and T93 from Rrs1 form intra-molecular hydrogen bonds. These three residues are labeled and displayed as yellow stick models. (**D**) Sequences in the proline-rich loop after CLUSTALW alignment (30). The sequences are as follows: An; *Aspergillus nidulans*, Sc; *Saccharomyces cerevisiae*, Hs; *Homo sapiens*, Rn; *Rattus norvegicus*, Ca; *Candida albicans*, At; *Arabidopsis thaliana*. The most conserved sites are highlighted in black. Red arrow indicates the region for which the structure was determined. (**E**) Close-up view of hydrogen-bonding interactions at the C-terminal loop of Rrs1. Amino acid residues that form the interaction are displayed as stick models. Conserved residues are denoted in Italics.

The two proteins were so intimately connected that they resembled a single molecule. Three regions contributed to the molecular interactions between Rpf2 and Rrs1. These were the long α-helix (α1), β-sheets and C-terminal proline-rich loop (residues 88–104) of Rrs1. The amphiphilic long α-helix of Rrs1 formed both hydrophobic and hydrophilic interactions with the wide β-sheet of Rpf2. A hydrophobic surface was formed by seven residues (L56, A60, V64, L67, L68, L71 and L72) on the α-helix of Rrs1. These residues were involved in hydrophobic interactions with the hydrophobic surface created by ten residues in Rpf2 (L145, L147, L185, A188, M190, I211, L220, P221, V223 and L225) (Figure 2A). Most of these residues are conserved across eukaryotes (Supplementary Figures S1 and S2). The hydrophilic surface of the Rrs1 α-helix was formed by five residues (N53, K57, R61, Q65 and N69). The side chains of K57, R61 and Q65 of Rrs1 formed hydrogen bonds with three main-chain oxygens of Rpf2 (P221, V223 and L225) (Figure 2B). The side-chain of Q65 formed an additional hydrogen bond with the amide nitrogen of V223 on a β-sheet of Rpf2. A hydrogen-bonded side chain interaction was observed between the N53 of Rrs1 and R207 of Rpf2 (Figure 2B). The N69 of Rrs1 also formed an intra-molecular hydrogen bond with I21 on the N-terminal loop.

Two β-strands (β1, β2) of Rrs1 joined the central β-sheet of Rpf2 to complete the 12-stranded β-barrel structure and the other two β-strands (β3, β4) formed a three-stranded β-sheet with the β7 strand of Rpf2 (Figure 1C).

The long C-terminal loop of Rrs1 wrapped around the side of Rpf2 (Figure 1A). This loop is highly conserved in eukaryotes; among the 17 residues in the loop (88–104), nine (L88, P89, P91, T93, L95, P96, R97, K99 and P102) are completely conserved. This loop is rich in proline residues that are either completely conserved (shown above) or highly conserved (P100 and P104) (Figure 2D, Supplementary Figure S1). Most of the conserved residues are involved in either intra- or inter-molecular interactions. The T93 of Rrs1 forms intra-molecular hydrogen bonds with the side-chain of D32 (Figure 2C). Three residues (P96, R97 and K99) of Rrs1 form hydrogen bonds with three main-chain carbonyl oxygens (Q22, L23 and A250) and three conserved side chains (E25, K28 and D84) of Rpf2 (Figure 2E, Supplementary Figure S2). These conserved interactions between Rpf2 and Rrs1 suggest the importance of the proline-rich loop. This conserved proline rich loop likely plays a role in locating the C-terminal domain of Rrs1 at the opposite side of the N-terminal domain with respect to Rpf2.

### 5S rRNA binding assay for Rpf2-Rrs1 complex

**Figure 3.**
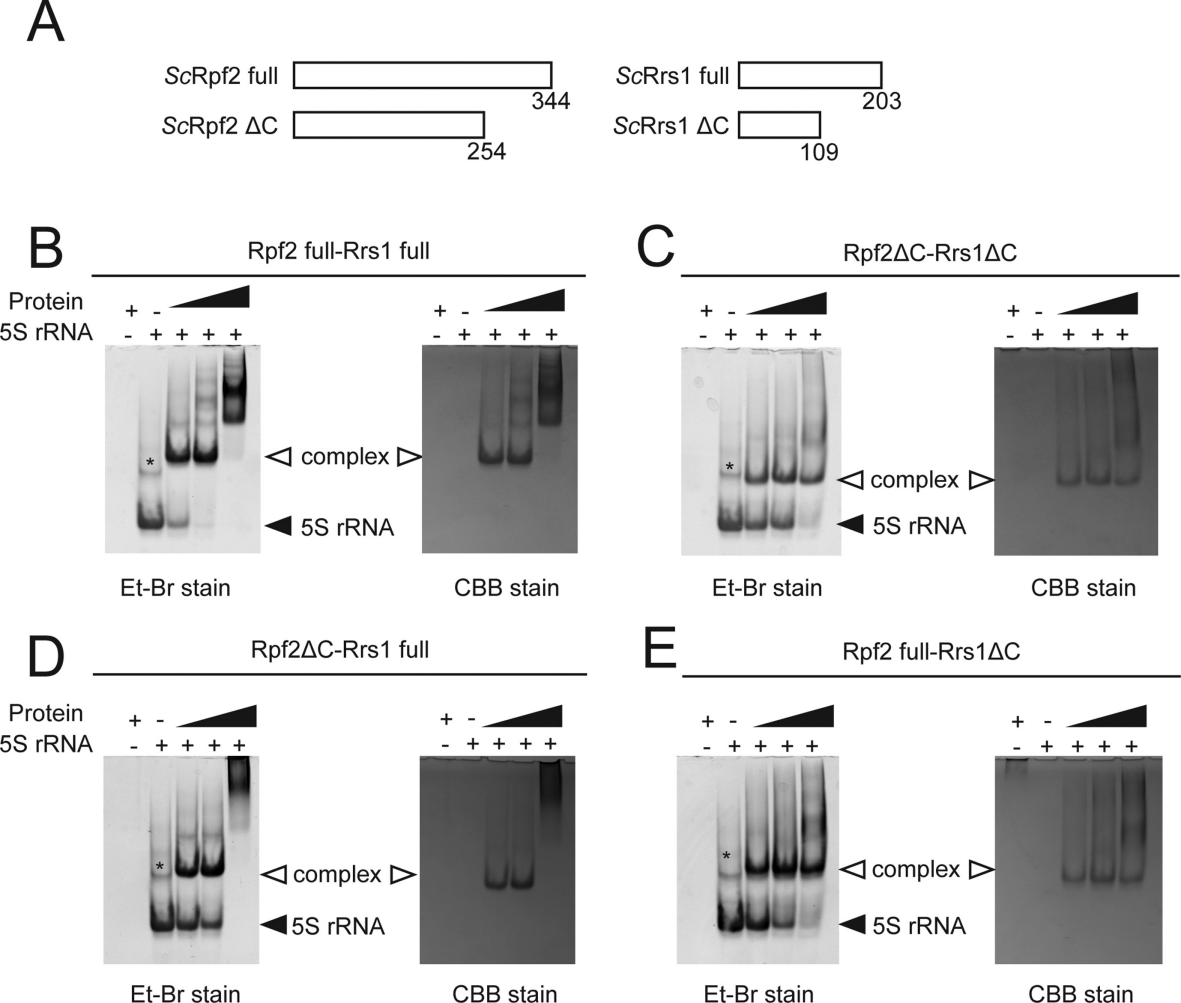
5S rRNA binding assay. (**A**) Schematic overview of the Rpf2 and Rrs1 variants. (**B**–**E**) Results of the gel shift assay. *Sc*5S rRNA (50 pmol) was incubated without factor or with 50, 100 and 200 pmol of *Sc*Rpf2-Rrs1 complex variants: *Sc*Rpf2 full-Rrs1 full (B), *Sc*Rpf2ΔC-Rrs1ΔC (C), *Sc*Rpf2ΔC-Rrs1 full (D), *Sc*Rpf2 full-Rrs1ΔC (E). Asterisk (*) indicates the 5S rRNA dimer, as confirmed by a gel-filtration analysis and urea-PAGE. Each gel was stained with ethidium bromide (Et-Br stain, left) and Coomassie Brilliant Blue (CBB stain, right).

The N-terminal domain of Rpf2 (residues 19–254) included the Brix domain (residues 28–245) (Figure 1B). As this domain is known as an RNA binding domain, it can be inferred that the Rpf2-Rrs1 complex contains a site for 5S rRNA binding. To confirm this functionality, a gel shift assay was performed in the presence of increasing amounts of Rpf2-Rrs1 complexes that had been pre-incubated with 5S rRNA. Because eukaryotic ribosome biogenesis has been studied most extensively in yeast, we used *S. cerevisiae* proteins and RNA for this experiment. The sequences of Rpf2 and Rrs1 are highly conserved between *A. nidulans* and *S. cerevisiae* (sequence identities of Rpf2 and Rrs1: 38.24% and 41.45%, respectively). As the amount of Rpf2-Rrs1 increased, bands corresponding to the 5S rRNA disappeared and bands corresponding to the Rpf2-Rrs1–5S rRNA complex appeared. This result indicated that the Rpf2-Rrs1 complex could bind to 5S rRNA (Figure 3B).

To characterize the binding feature, we used three C-terminal deletion complexes (*Sc*Rpf2ΔC-Rrs1, *Sc*Rpf2-Rrs1ΔC, *Sc*Rpf2ΔC-Rrs1ΔC) in binding experiments (Figure 3A). All deletion complexes exhibited the 5S rRNA complex bands observed with the full-length Rpf2-Rrs1 complex (Figure 3C–E). These results revealed that the N-terminal core complex alone could bind 5S rRNA. However, as the bands for 5S rRNA were present even under the highest concentration of proteins, the C-terminal domains also appear to be involved in 5S rRNA binding. For the *Sc*Rpf2-Rrs1 and *Sc*Rpf2ΔC-Rrs1 complexes, an extra band at a higher molecular weight than the 5S rRNA complex was also observed at a higher protein concentration (Figure 3B and D); however, these bands were not detected with the *Sc*Rpf2-Rrs1ΔC complex, suggesting that the C-terminal domain of Rrs1 was involved in oligomerization.

### Structural elements involved in binding between the Rpf2-Rrs1 complex and 5S rRNA

**Figure 4.**
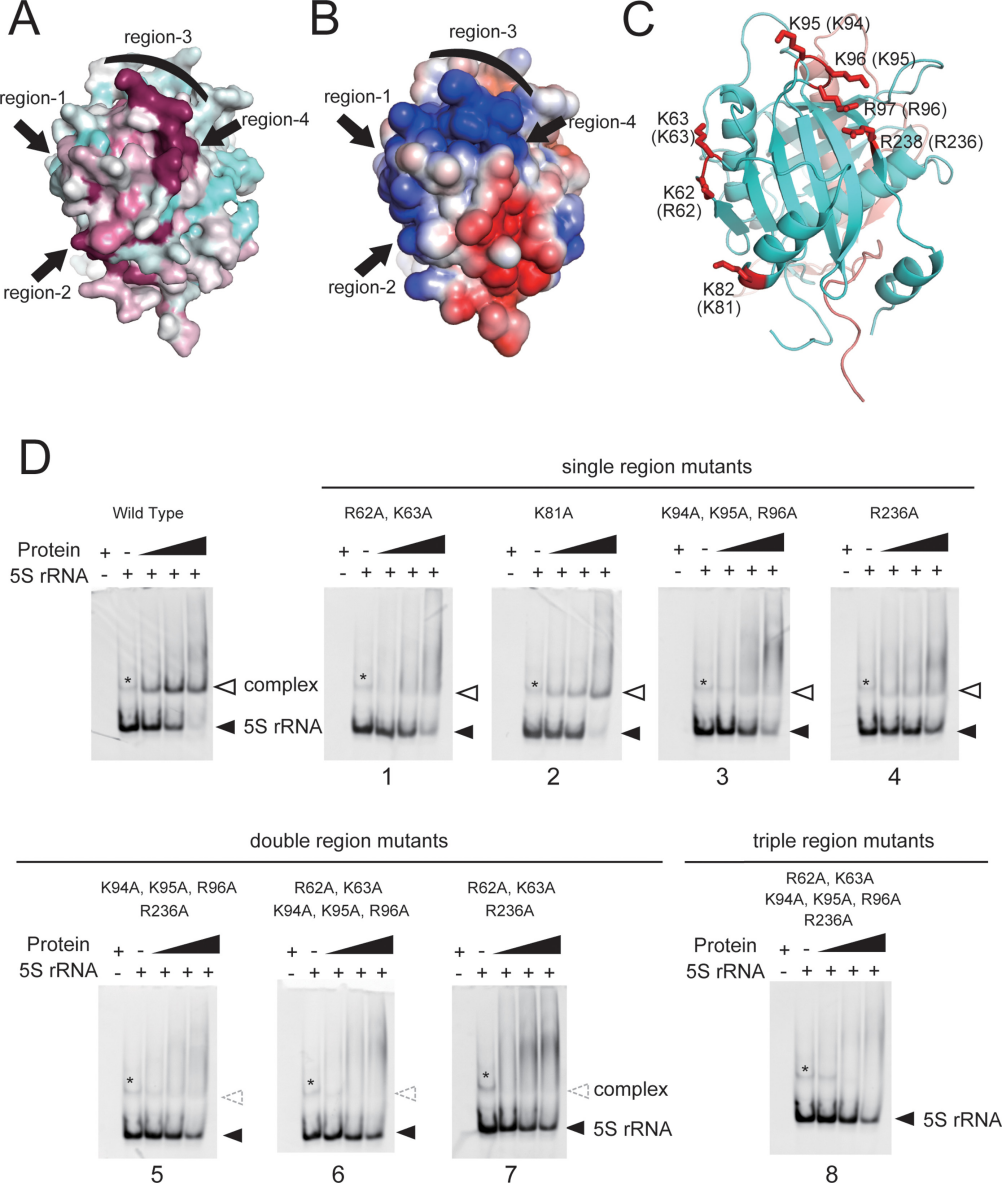
Evolutionarily conserved and electrically positive regions of *Sc*Rpf2 (region-1 to 4) were selected for mutagenesis experiments to test the significance of the interactions with 5S rRNA. (A) Sequence conservation is mapped onto the surface along with variable (cyan) and conserved (purple) residues using Consurf (28). (B) Electrostatic surface potential diagrams with positive (blue) and negative (red) electrostatic potentials are mapped onto a van der Waals surface diagram of the conserved surface patch using APBS (29) The color scale ranges between −3 *k_B_T* (red) and +3 *k_B_T* (blue), where *k_B_* is Boltzmann's constant and *T* is temperature. (**C**) Ribbon diagram of the Rpf2-Rrs1 core complex in the same orientation as in A and B. Four regions containing seven residues (red) were selected for mutation analysis. Residue numbers of *Sc*Rpf2 are shown. Letters in parentheses correspond to the residue numbers for *An*Rpf2. (**D**) Results of the gel shift assay. *Sc*5S rRNA (50 pmol) was incubated without factor or with 50, 100, or 200 pmol of *Sc*Rpf2-Rrs1 complex mutated variants; the denoted numbers correspond to the mutant No. (Supplementary Table S1). Wild type indicates *Sc*Rpf2ΔC-Rrs1ΔC purified using the same method used to purify other point-mutated variants. Asterisk (*) indicates the 5S rRNA dimer, as confirmed by a gel-filtration analysis and urea-PAGE. Results are shown as an ethidium bromide-stained gel.

**Figure 5.**
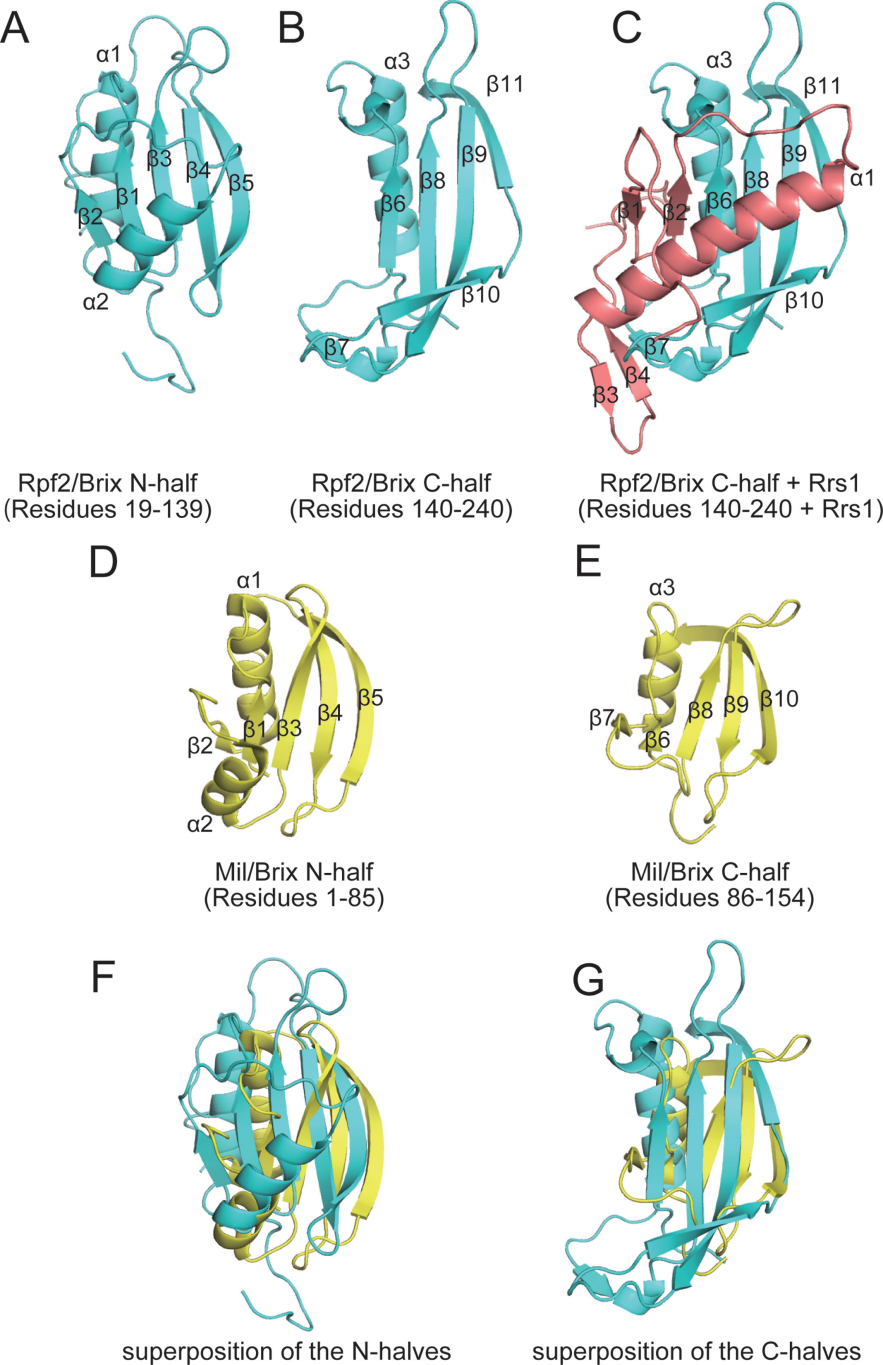
Structural comparison of the N- and C-terminal halves of the Brix domains. (**A** and **B**) N- and C-terminal halves of Rpf2/Brix domain and (**C**) C-terminal half of Rpf2/Brix domain plus Rrs1. The long α-helix of Rrs1 appears to correspond with the C-terminal half of the Rpf2/Brix domain. (**D** and **E**) The N- and C-terminal halves of Mil/Brix, showing the duplicated architecture. (**F** and **G**) The superposition of the N-halves and C-halves of Rpf2/Brix and Mil/Brix. Rpf2, Rrs1 and Mil are colored in cyan, pink and yellow, respectively.

**Figure 6.**
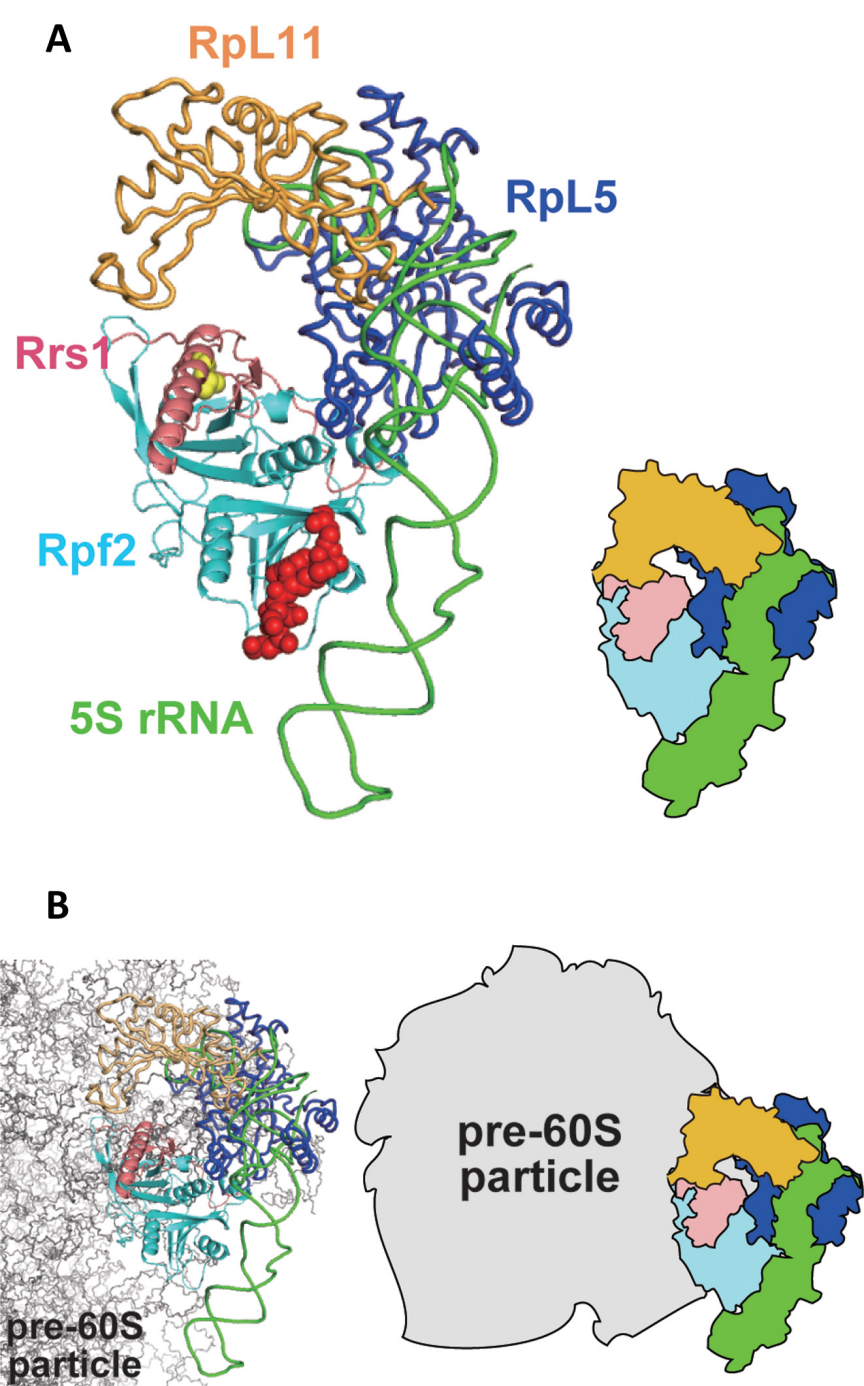
Rpf2-subcomplex model and its location on the ribosomal precursor. (**A**) Rpf2-subcomplex model (left) and schematic representation (right). Red spheres represent the 5S rRNA binding region on Rpf2. Yellow spheres represent the RpL5 and RpL11 interaction region on Rrs1. (**B**) Rpf2-subcomplex model superposed on the ribosomal precursor (left); schematic representation (right). The molecules are colored as follows: cyan, Rpf2; pink, Rrs1; green, 5S rRNA; light orange, RpL11; blue, RpL5; gray, pre-60S particle.

A detailed inspection of the complex surface in terms of sequence conservation and surface potentials highlighted four possible regions for 5S rRNA binding on Rpf2 (Figure 4A and B, Supplementary Figure S2). Based on these results, we prepared various mutation variants of these regions and inspected the 5S rRNA binding abilities (Figure 4C, Supplementary Table S1). The mutational analysis was performed on *S. cerevisiae* Rpf2. Thus, in this section, we described residue numbers of *S. cerevisiae* Rpf2 first and *A. nidulans* Rpf2 in parentheses. The substitution of alanine for all residues in region-1 (R62, K63 (K62, K63)), region-3 (K94, K95 and R96 (K95, K96 and R97)) and region-4 (R236 (R238)) partially disrupted the binding to 5S rRNA (Figure 4D-1, 3, 4). On the other hand, no effect was detected for region-2 (K81 (K82)) mutants (Figure 4D-2). A combined region-3 and -4 mutation drastically affected the interaction with 5S rRNA (Figure 4D-5). The other two combination mutants yielded similar results (Figure 4D-6, 7). A triple-region mutation completely disrupted binding to 5S rRNA (Figure 4D-8), indicating that R62, K63, K94, K95, R96 and R236 are important residues for the interaction between Rpf2-Rrs1 and 5S rRNA. Among these, the R236 (R238) might be most important for the interaction with 5S rRNA, as a single amino acid mutation significantly reduced the binding ability. Indeed, this residue is located on a σ^70^-like motif that is reportedly involved in RNA binding (10)(Supplementary Figure S2). Moreover, a cation-π interaction was observed between R236 and F69 (R238 and F70) in our structure (Supplementary Figure S3-A). Alanine mutations of the conserved residues P68, F69 and E70 (P69, F70 and E71) disrupted binding to 5S rRNA (Supplementary Figures S2 and S3-B), although its tertiary structure did not significantly differ from the wild type as detected from the CD spectra (Supplementary Figure S3-C).

## DISCUSSION

### Structural aspect of full-length Rpf2-Rrs1 complex

In this study, we reported the crystal structure of the *An* Rpf2-Rrs1 core complex (Rpf2 [residues 18–262]-Rrs1 [residues 10–113]). This is the core region of the Rpf2-Rrs1 complex as suggested by its resistance against proteolytic digestion (22). The structural analysis revealed that these N-terminal domains of Rpf2 and Rrs1 bind each other tightly. PSIPRED programs for predicting secondary structures (34) suggested that the C-terminal region of Rpf2 is a flexible loop (approximately 80 residues). On the other hand, the C-terminal region of Rrs1 was predicted to have a secondary structure, suggesting that the intact Rrs1 comprises two similarly sized domains connected by a central proline-rich linker region (residues 88–108). Therefore, the intact Rpf2 and Rrs1 molecules appear to work with the anchoring core domains of the N-terminal regions as well as the peripheral C-terminal regions.

### Rpf2/Brix domain as a highly evolutionarily modified scaffold domain

The Rpf2/Brix domain includes a half-β-barrel surrounded by three α-helices and two 3_10_ turn helices. The N- and C-terminal halves of the Brix domain are thought to have evolved via gene duplication. Indeed, the N- and C-terminal halves of Mil exhibited a similar architecture (Figure 5D and E) (14). In the Rpf2/Brix domain, although the folding topologies were conserved between the N- (residues 19–139) and C- (residues 140–240) terminal halves, the appearances of the two structures were extensively divergent (Figure 5A and B). Interestingly, the long α-helix of Rrs1 appeared to join the C-terminal half of Rpf2/Brix domain; the α1 and β2 of Rrs1 correspond to α2 and β2 of the N-terminal half of the Rpf2/Brix domain, respectively (Figure 5A and C). These structural features suggest that the C-terminal half of the Rpf2/Brix domain underwent extensive evolution after gene duplication through the addition of extra regions and replacement of the α-helix with that of Rrs1.

The Brix domain contains a σ^70^-like RNA binding motif between β10 and β11 (*An*Rpf2: 223–240) (10) (Supplementary Figure S2). The structure of the Rpf2-Rrs1 core complex demonstrated that this motif comprises a sheet-turn-sheet structure and is located at the center of the Brix domain. The finding that the R238 on this motif was significant for 5S rRNA binding (as mentioned above) is consistent with the findings of a previous study (10). In addition, V223 and L225 on this motif were shown to be involved in binding to Rrs1, suggesting that this motif includes an important region for binding both RNA and proteins.

The Brix domain is known as a scaffold domain. Each protein containing this domain is associated with a specific partner. The present study showed that the N-terminal half of the Rpf2/Brix domain bound to 5S rRNA and the C-terminal half bound to Rrs1. However, R220 and R253 in C-terminal half of the Imp4/Brix domain (*S. cerevisiae*) has been reported as important for the association with U3 snoRNA (35). Therefore, the Brix domain has undergone significant specialization through evolution while retaining its core structure.

### Role of Rrs1 and the proline-rich linker

The N-terminal domain of *An*Rrs1 (residues 19–104), which was determined in the present study, is closely related to the cold-sensitive mutant *rrs1–1* of *Sc*Rrs1 (residues 1–113). Although *rrs1–1* causes a defect in rRNA processing at low temperatures, it is viable at temperatures higher than 25ºC (15,20), suggesting that the N-terminal domain of Rrs1 itself plays a significant role in its function. Our present results showed that the N-terminal domain of Rrs1 played a role in ribosome biogenesis via interaction with the N-terminal domain of Rpf2. In addition, yeast-two hybrid assays showed that the *Sc*Rrs1 L65P mutant inhibited the associations with two ribosomal proteins (RpL5 and RpL11) (21). This residue corresponds to L71 of *An*Rrs1 and is located on the C-terminal portion of the long α-helix. As proline has been noted as α-helix breaker, the substitution of proline for leucine in the α-helix caused partial disruption of the C-terminal region of the helix. Consequently, Rrs1 might have lost its capacity to bind RpL5 and RpL11. Taken together, these facts suggest that the N-terminal domain of Rrs1 is an important adaptor domain for the interactions with Rpf2, RpL5 and RpL11.

It is generally accepted that eukaryotic inter-domain linkers have rather tight structures (e.g. helical or proline-rich structures) and act as rigid spacers to prevent unfavorable interactions between two domains (36). Actually, the deletion or shortening of linker segments sometimes prohibits their function (37–39). Similarly, in Rrs1, the linker region connecting the N- and C-terminal domains comprises a conserved proline-rich segment (88–107) (Supplementary Figure S1). This chain segment runs along the molecular surface of Rpf2 (Figure 2C). Highly specific interactions were observed between the conserved residues at the ending position of the linker and the conserved residues of Rpf2 (Figure 2E). A mutant (*An*Rrs1 residues 1–90) lacking this region retained the capacity to bind Rpf2 (data not shown), suggesting that these conserved interactions are not required for the binding of two N-terminal domains. Therefore, it is likely that these conserved interactions are used to anchor the terminal region of the linker at a specific position on Rpf2. These facts suggest that the C-terminal domain of the Rrs1 linker localizes on a specific Rpf2 site (i.e. a site remote from the N-terminal domain of Rrs1). The results of a binding assay with 5S rRNA indicated that Rpf2 full-Rrs1ΔC had a lower binding affinity for 5S rRNA relative to Rpf2 full-Rrs1 full, indicating that the Rrs1 C-terminal domain assists in the interaction with 5S rRNA. Taken together, the N- and C-terminal domains of Rrs1 are arranged on separate positions of Rpf2 via a proline-rich linker and play distinct roles in ribosome biogenesis.

### Binding model of Rpf2-subcomplex with 90S ribosome

The structural analysis of the Rpf2-Rrs1 complex together with a previous functional analysis suggested that the C-terminal part of the long α-helix of Rrs1 associates with RpL5 and RpL11 and that the conserved basic patch on Rpf2 recognizes 5S rRNA. As the eukaryotic ribosomal structure has been analyzed and the binding manners of 5S rRNA, RpL5 and RpL11 are known (40), we attempted to construct a complex model containing five molecules (Rpf2-subcomplex) by docking the Rpf2-Rrs1 core complex on the tripartite complex (5S rRNA-RpL5-RpL11). For this model building, the Rrs1 region (the C-terminal portion of the long α-helix) that binds with RpL5 and RpL11 as has been explained in section ‘Role of Rrs1 and the proline-rich linker’ was placed in the vicinity of RpL5 and RpL11 in the 5S rRNA-RpL5-RpL11 complex. Furthermore, the Rpf2 basic patch that binds with 5S rRNA was placed close to 5S rRNA in the 5S rRNA-RpL5-RpL11 complex. In this model, Rpf2-Rrs1 was snugly situated at the inside of the curved region of 5S rRNA (Figure 6A). This Rpf2-subcomplex model satisfied the results of the binding assay that revealed direct interactions within each pair formed by the molecules Rrs1, Rpf2, RpL5 and RpL11 (17–21). In addition, in the model both the C-termini of Rpf2 and Rrs1 (Rpf2: G254, Rrs1: P104) were positioned at the 5S rRNA side. This observation accounted for the results of the gel shift assay involving C-terminal deletion complexes, which suggested that both C-terminal domains were involved to some extent in the interactions with 5S rRNA.

Rpf2 and Rrs1 are localized on 90S and pre-60S particles in the nucleolus and are involved in 27SB rRNA processing (10,18). Although the structure of the nuclear ribosome precursor has not yet been determined, a recent cryo-electron microscopy study revealed the structure of the cytoplasmic pre-60S particle in which 5S rRNA is rotated nearly 180º relative to the mature subunit (PDB code: 4V7F) (41). The Rpf2-subcomplex model was superposed on the 5S rRNA-RpL5-RpL11 of this pre-60S particle, suggesting that the Rpf2-Rrs1 complex is located between 5S rRNA and the ribosome precursor (Figure 6B). This indicates that the Rpf2-Rrs1 complex can associate directly with the 90S precursor even after Rpf2-subcomplex formation. Furthermore, this model demonstrates that a wide, positively charged region of Rpf2 faces the ribosome precursor and that the Rpf2-Rrs1 complex occupies the 25S rRNA binding regions of RpL5 and RpL11, suggesting that instead of these proteins, the Rpf2-Rrs1 complex might associate with pre-rRNA on the 90S precursor. For more detailed discussion, we must await the structure analysis of Rpf2-subcomplex.

## ACCESSION NUMBERS

Atomic coordinates and structure factors have been deposited to the Protein Data Bank under the accession number 4XD9.

## SUPPLEMENTARY DATA

Supplementary Data are available at NAR Online.

SUPPLEMENTARY DATA
