# Supplementary material for: Structural and functional analysis of the Rpf2-Rrs1 complex in ribosome biogenesis
Source: Nucleic Acids Res. 2015 Apr 8;43(9):4746–57. doi: 10.1093/nar/gkv305 (PMC4482071; doi:10.1093/nar/gkv305)
Supplement: SUPPLEMENTARY DATA [file supp_43_9_4746__index.html]

Structural and functional analysis of the Rpf2-Rrs1 complex in ribosome biogenesis — Structural and functional analysis of the Rpf2-Rrs1 complex in ribosome biogenesis — SUPPLEMENTARY DATA 

# Structural and functional analysis of the Rpf2-Rrs1 complex in ribosome biogenesis

## SUPPLEMENTARY DATA

**Files in this Data Supplement:**

- SUPPLEMENTARY DATA
- SUPPLEMENTARY DATA
