# Supplementary material for: Structural and functional analysis of the Rpf2-Rrs1 complex in ribosome biogenesis
Source: Nucleic Acids Res. 2015 Apr 8;43(9):4746–57. doi: 10.1093/nar/gkv305 (PMC4482071; doi:10.1093/nar/gkv305)
Supplement: SUPPLEMENTARY DATA [file supp_gkv305_nar-00318-r-2015-File009.pdf]

Supplementary Tables

Supplementary Table S1. Summary of mutant construction

Mutant No.	Template	Primer	Mutation	Mutated region name
1	*Sc*Rpf2 ΔC	S1, AS1	R62A, K63A	region-1
2	*Sc*Rpf2 ΔC	S2, AS2	K81A	region-2
3	*Sc*Rpf2 ΔC	S3, AS3	K94A, K95A, R96A	region-3
4	*Sc*Rpf2 ΔC	S4, AS4	R236A	region-4
5	mutant 3	S4, AS4	K94A, K95A, R96A, R236A	
6	mutant 1	S3, AS3	R62A, K63A, K94A, K95A, R96A	
7	mutant 1	S4, AS4	R62A, K63A, R236A	
8	mutant 5	S1, AS1	R62A, K63A, K94A, K95A, R96A, R236A	
9	*Sc*Rpf2 ΔC	S5, AS5	P68A, F69A, E70A	

Supplementary Table S2. Primers used in this study

Primer No.	Sequence
S1	AATgcggcgAATGATATTCATCCTTTCGAAGACATGTC
S2	GAAgcgAATGACTGTTCATTGATGGTGCTGATG
S3	TCCgcggcggcgAAAAACAACATGACCTTTATACGTACATTTG
S4	GGCgcgATTCATACTCCAAGTCCAGATATGGTCACT
S5	CATgcggcggcgGACATGTCGCCACTAGAGTTCTTTAGTG
5S_S-1	AATTC**TAATACGACTCACTATA**GGTTGCGGCCATATCTACCAGAAA
5S_S-2	GCACCGTTTCCCGTCCGATCAACTGTAGTTAAGCTGGTAAGAGCCTGACC
5S_S-3	GAGTAGTGTAGTGGGTGACCATACGCGAAACTCAGGTGCTGCAGT*CGACG*
5S_S-4	*GAGTCTAGACTCCGTCCTGATGAGTCCGTG*
5S_S-5	*AGGACGAAACTGCAAAATAAACCAAGGATC*AA
5S_S-6	CCCCTTGGGGCCTCTAAACGGGTCTTGAGGGGTTTTTTGA
AS1	GAACCTCTTCATATCTGGCTTCTTCAAG
AS2	ACTAAAGAACTCTAGTGGCGACATGTCTTC
AS3	ACTTGTCATCAGCACCATCAATGAAC
AS4	GATTTTGAAATCTAGACGTGGCCCA
AS5	AATATCATTCTTACGATTGAACCTCTTCATATCT
5S_AS-1	ATATGGCCGCAACC**TATAGTGAGTCGTATTA**G
5S_AS-2	TTACCAGCTTAACTACAGTTGATCGGACGGGAAACGGTGCTTTCTGGTAG
5S_AS-3	AGCACCTGAGTTTCGCGTATGGTCACCCACTACACTACTCGGTCAGGCTC
5S_AS-4	*GTTTCGTCCTCACGGACTCATCAGGACGGAGTCTAGACTCCGTCG*ACTGC
5S_AS-5	CCCCAAGGGGTT*GATCCTTGGTTTATTTTGCA*
5S_AS-6	AGCTTCAAAAAACCCCTCAAGACCCGTTTAGAGG
5S_AS-7	AGATTGCAGCACCTTCACAGGCTCTTAC

The T7 promoter and terminator sequences are shown in bold. The 3' end of the ribozyme sequence is shown in italics. The mutation sequences are shown in lowercase.

Supplementary Table S3. Statistics of native and SeMet SAD data collection and structure refinement

	Native crystal	SeMet-labeled crystal
Wavelength (Å)	1.0000	0.9788
Space group	$P2_12_12_1$	$P4_12_12$
a, *b*, *c* (Å)	54.1, 123.3, 133.8	128.9, 128.9, 57.5
α, β, γ (°)	90, 90, 90	90, 90, 90
Resolution range (Å)	50 - 2.35 (2.49 - 2.35)	50-3.51(3.71-3.51)
Completeness (%)	99.4 (97.6)	99.9 (99.7)
Multiplicity	7.20 (7.26)	7.81 (7.56)
$I/\sigma(I)$	14.51 (2.57)	12.96 (1.95)
$^{a}R_{meas}$	9.1 (77.6)	15.7 (108.3)
**Refinement**		
Resolution range (Å)	42-2.35	
Reflections work/test	36222/1909	
R_{work} (%)	19.2	
$^{b}R_{free}$ (%)	23.5	
No. of atoms		
Protein	5002	
Solvent molecules	163	
RMS deviations		
Bond lengths (Å)	0.003	
Angles (deg)	0.910	

Values in parentheses are for the highest resolution shell.

$^{a}R_{meas} = \sum_{hkl}\{N(hkl)/[N(hkl) - 1]\}^{1/2}\sum_i|I_i(hkl) - \langle I(hkl)\rangle|/\sum_{hkl}\sum_i I_i(hkl)$, where $\langle I(hkl)\rangle$ and $N(hkl)$ are the mean intensity of a set of equivalent reflections and the multiplicity, respectively.

$^{b}R_{free}$ was calculated from 5% of the randomly selected reflections that were excluded from the refinement.

Supplementary Figures

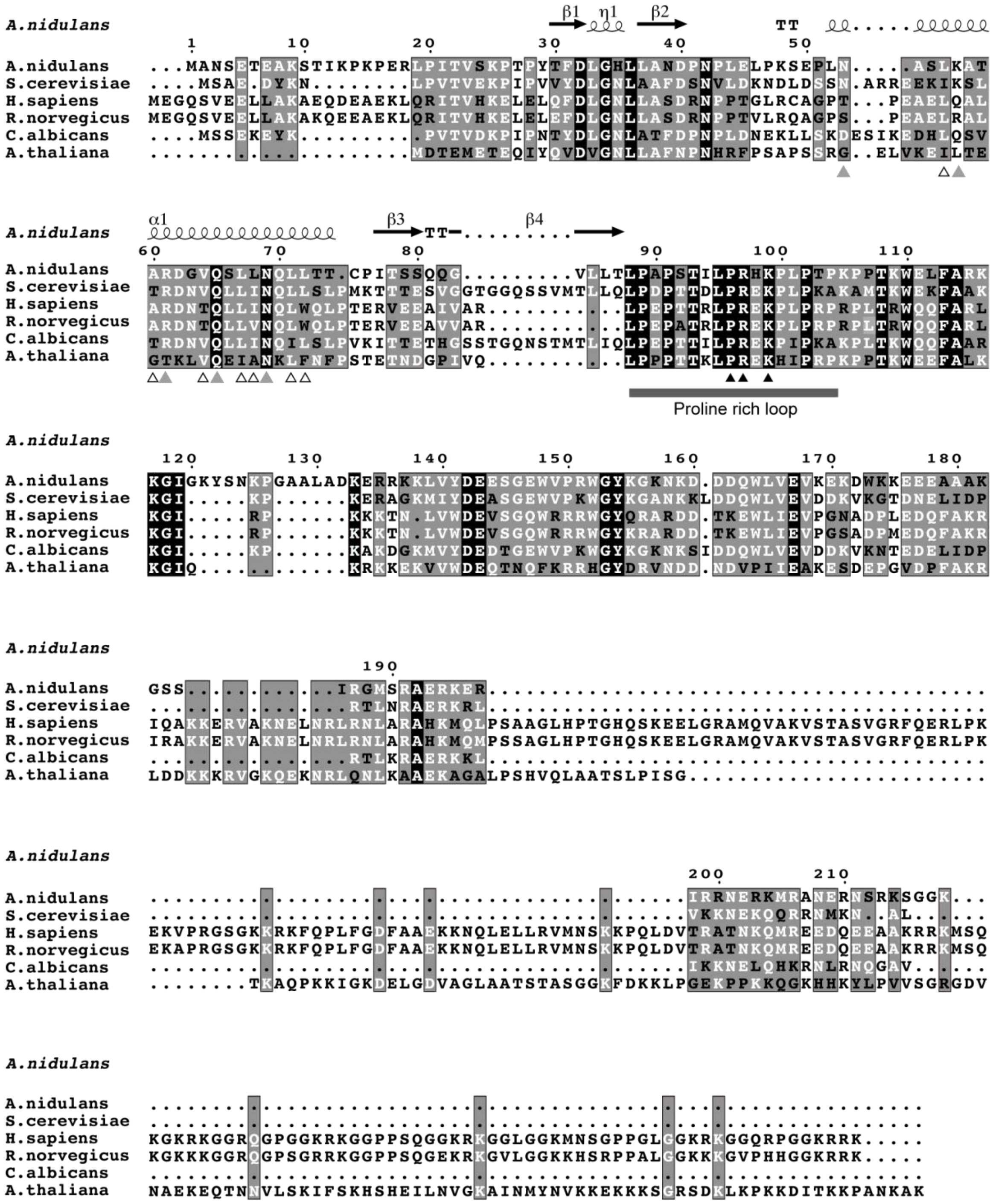

Figure S1

Rrs1 amino acid sequence alignment. The sequences displayed are as follows: A. nidulans, *Aspergillus nidulans*; S. cerevisiae, *Saccharomyces cerevisiae*; H. sapiens, *Homo sapiens*; R. norvegicus, *Rattus norvegicus*; C. albicans, *Candida albicans*; A. thaliana, *Arabidopsis thaliana*. The most conserved sites are highlighted in black. Each protein sequence was aligned using CLUSTALW and the figure was prepared with the program ESPript. Secondary structure elements of the *A.*

nidulans Rrs1 N-terminal domain are also shown. The residues interacted with Rpf2 are marked by the following symbols: white triangle, hydrophobic interactions on long α-helix of Rrs1; gray triangle, hydrogen-bond on long α-helix of Rrs1; black triangle, interactions on C-terminal proline-rich loop of Rrs1.

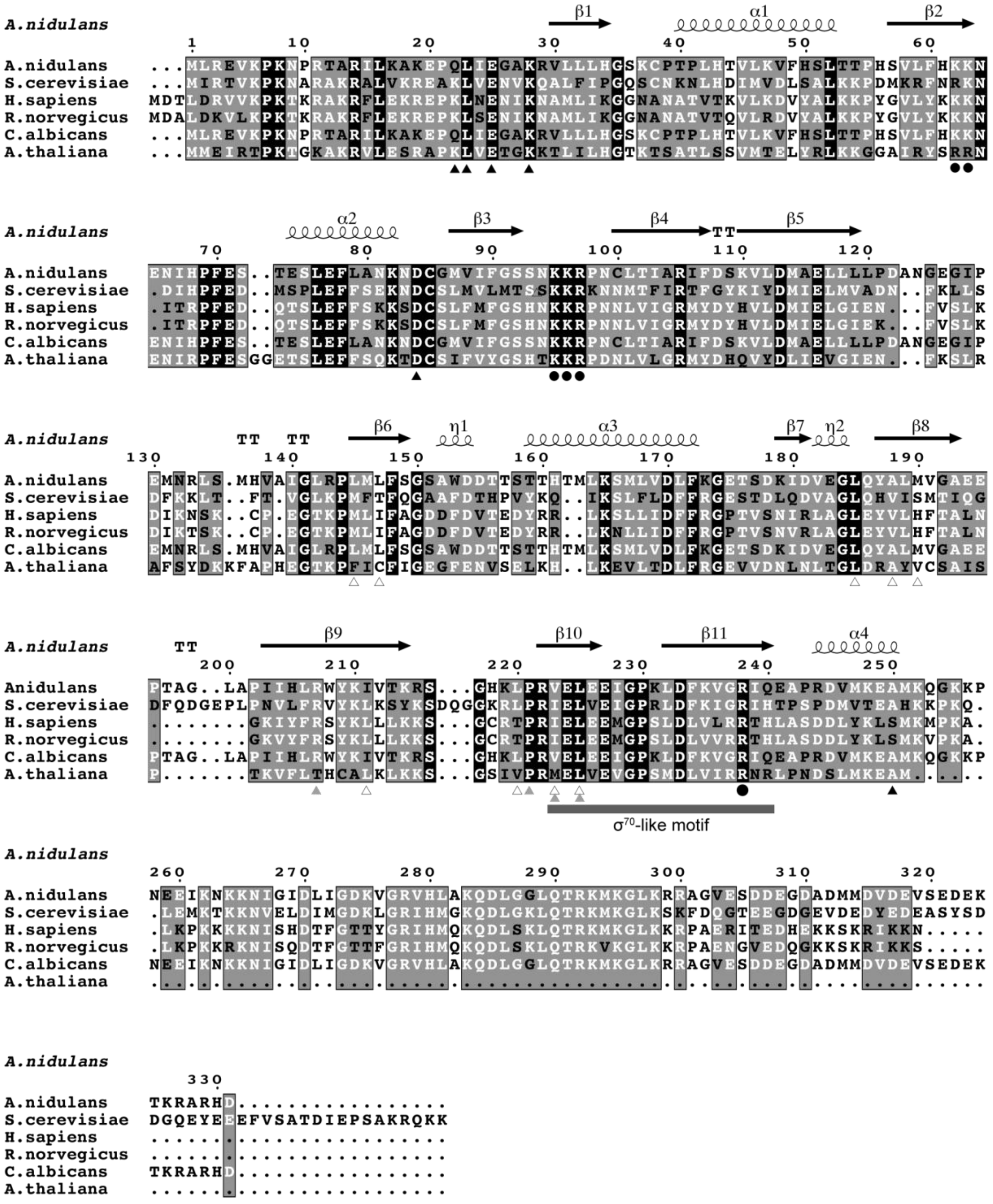

Figure S2

Rpf2 amino acid sequence alignment. The sequences displayed are as follows: A. nidulans, *Aspergillus nidulans*; S. cerevisiae, *Saccharomyces cerevisiae*; H. sapiens, *Homo sapiens*; R. norvegicus, *Rattus norvegicus*; C. albicans, *Candida albicans*; A. thaliana, *Arabidopsis thaliana*. The most conserved sites are highlighted in black. Each protein sequence was aligned using CLUSTALW and the figure was prepared with the program ESPript. Secondary structure elements of the *A. nidulans* Rpf2 N-terminal domain are also shown. The residues interacted with Rrs1 are marked by the following symbols: white triangle, hydrophobic interactions with long α-helix of Rrs1; gray triangle,

hydrogen-bonds with long α-helix of Rrs1; black triangle, interactions with C-terminal proline-rich loop of Rrs1.

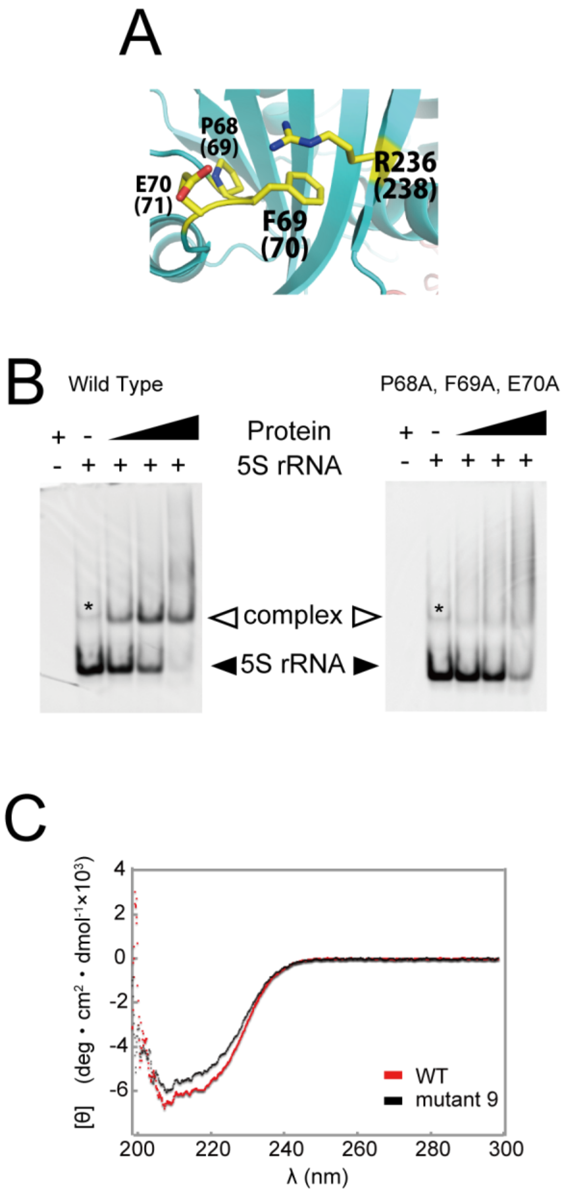

Figure S3

(A-C) The cation-π interaction between F69 and R236 of *Sc*Rpf2 and results of alanine mutations around F70. (A) Close view of the contact between F236 and F69. Letters in parentheses correspond to residue numbers for *An*Rpf2. (B) Results of a gel shift assay with wild type (left) or mutant 9 (right), in which alanine substitutes for P68, F69 and E70. *Sc*5S rRNA (50 pmol) was incubated without factor or with 50, 100 and 200 pmol of *Sc*Rpf2-Rrs1 complex. Wild type indicates *Sc*Rpf2ΔC-Rrs1ΔC purified using same method used to purify other point-mutated variants. Asterisk (*) indicates the 5S rRNA dimer, as confirmed by a gel-filtration analysis and urea-PAGE. (C) CD spectra of the *Sc*Rpf2-Rrs1 complex. Red line; wild type, Black line; mutant 9.
