# Supplementary material for: Structural and functional analysis of the Rpf2-Rrs1 complex in ribosome biogenesis
Source: Nucleic Acids Res. 2015 Apr 8;43(9):4746–57. doi: 10.1093/nar/gkv305 (PMC4482071; doi:10.1093/nar/gkv305)
Supplement: SUPPLEMENTARY DATA [file supp_gkv305_nar-00318-r-2015-File010.pdf]

Preliminary wwPDB X-ray Structure Validation Summary Report (i)

Dec 19, 2014 – 12:32 AM EST

This is a Preliminary wwPDB validation summary report for a publicly released PDB entry.
This report is produced by the wwPDB validation pipeline
before deposition or annotation of the structure.
This is not an official wwPDB validation report and is not a proof of deposition.
This report should not be submitted to journals.
We welcome your comments at validation@mail.wwpdb.org
A user guide is available at http://wwpdb.org/ValidationPDFNotes.html

The following versions of software and data (see references) were used in the production of this report:

MolProbity	:	4.02b-467
Mogul	:	1.16 November 2013
Xtriage (Phenix)	:	dev-1439
EDS	:	stable24103
Percentile statistics	:	21963
Refmac	:	5.8.0049
CCP4	:	6.3.0 (Settle)
Ideal geometry (proteins)	:	Engh & Huber (2001)
Ideal geometry (DNA, RNA)	:	Parkinson et. al. (1996)
Validation Pipeline (wwPDB-VP)	:	stable24103

1 Overall quality at a glance ⓘ

The reported resolution of this entry is 2.35 Å.

Percentile scores (ranging between 0-100) for global validation metrics of the entry are shown in the following graphic. The table shows the number of entries on which the scores are based.

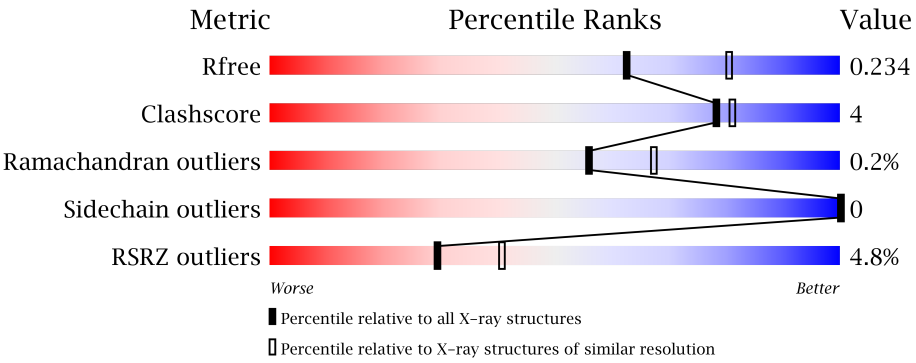

Metric	Whole archive (#Entries)	Similar resolution (#Entries, resolution range(Å))
R_{free}	66092	4049 (2.38-2.30)
Clashscore	79885	1094 (2.36-2.32)
Ramachandran outliers	78287	1080 (2.36-2.32)
Sidechain outliers	78261	1081 (2.36-2.32)
RSRZ outliers	66119	4050 (2.38-2.30)

The table below summarises the geometric issues observed across the polymeric chains and their fit to the electron density. The red, orange, yellow and green segments on the lower bar indicate the fraction of residues that contain outliers for >=3, 2, 1 and 0 types of geometric quality criteria. The upper red bar (where present) indicates the fraction of residues that have poor fit to the electron density.

Mol	Chain	Length	Quality of chain
1	D	86	
2	A	236	
2	C	236	
3	B	88	

WORLDWIDE PDB PROTEIN DATA BANK

2 Entry composition (i)

There are 4 unique types of molecules in this entry. The entry contains 5165 atoms, of which 0 are hydrogen and 0 are deuterium.

In the tables below, the ZeroOcc column contains the number of atoms modelled with zero occupancy, the AltConf column contains the number of residues with at least one atom in alternate conformation and the Trace column contains the number of residues modelled with at most 2 atoms.

- Molecule 1 is a protein.

Mol	Chain	Residues	Atoms					ZeroOcc	AltConf	Trace
			Total	C	N	O	S			
1	D	86	645	414	108	122	1	0	0	0

- Molecule 2 is a protein.

Mol	Chain	Residues	Atoms					ZeroOcc	AltConf	Trace
			Total	C	N	O	S			
2	A	236	1846	1176	320	337	13	0	0	0
			Total	C	N	O	S			
2	C	236	1846	1176	320	337	13	0	0	0

- Molecule 3 is a protein.

Mol	Chain	Residues	Atoms					ZeroOcc	AltConf	Trace
			Total	C	N	O	S			
3	B	88	665	425	113	126	1	0	0	0

- Molecule 4 is water.

Mol	Chain	Residues	Atoms		ZeroOcc	AltConf
			Total	O		
4	S	163	163	163	0	0

3 Residue-property plots (i)

These plots are drawn for all protein, RNA and DNA chains in the entry. The first graphic for a chain summarises the proportions of errors displayed in the second graphic. The second graphic shows the sequence view annotated by issues in geometry and electron density. Residues are color-coded according to the number of geometric quality criteria for which they contain at least one outlier: green = 0, yellow = 1, orange = 2 and red = 3 or more. A red dot above a residue indicates a poor fit to the electron density (RSRZ > 2). Stretches of 2 or more consecutive residues without any outlier are shown as a green connector. Residues present in the sample, but not in the model, are shown in grey.

- Molecule 1:

Chain D: 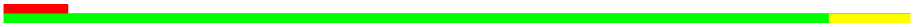

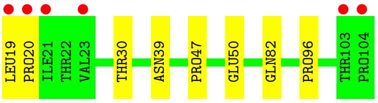

- Molecule 2:

Chain A: 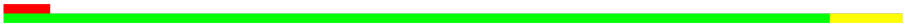

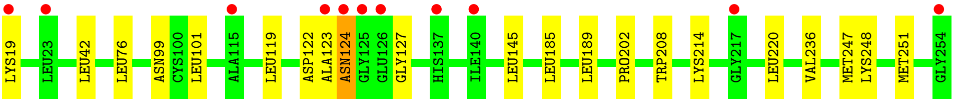

- Molecule 2:

Chain C: 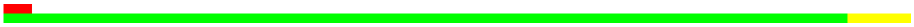

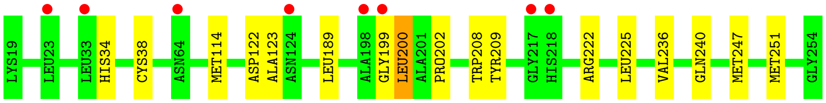

- Molecule 3:

Chain B: 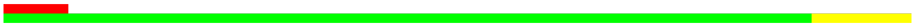

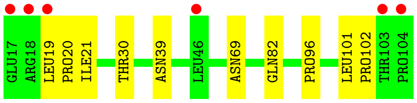

4 Data and refinement statistics (i)

Property	Value	Source
Space group	P 21 21 21	Depositor
Cell constants<br>a, b, c, α, β, γ	54.16Å 123.59Å 133.82Å<br>90.00° 90.00° 90.00°	Depositor
Resolution (Å)	42.10 – 2.35<br>46.51 – 2.35	Depositor<br>EDS
% Data completeness<br>(in resolution range)	99.4 (42.10-2.35)<br>99.5 (46.51-2.35)	Depositor<br>EDS
R_{merge}	(Not available)	Depositor
R_{sym}	(Not available)	Depositor
$< I/\sigma(I) >$ [1]	1.59 (at 2.34Å)	Xtriage
Refinement program	PHENIX (phenix.refine: 1.9_1692)	Depositor
R, R_{free}	0.192 , 0.235<br>0.192 , 0.234	Depositor<br>DCC
R_{free} test set	1910 reflections (5.01%)	DCC
Wilson B-factor (Å^2)	49.0	Xtriage
Anisotropy	0.100	Xtriage
Bulk solvent k_{sol}(e/Å^3), B_{sol}(Å^2)	0.35 , 50.8	EDS
Estimated twinning fraction	No twinning to report.	Xtriage
L-test for twinning	$< \|L\| > = 0.47$, $< L^2 > = 0.30$	Xtriage
Outliers	0 of 38135 reflections	Xtriage
F_o,F_c correlation	0.95	EDS
Total number of atoms	5165	wwPDB-VP
Average B, all atoms (Å^2)	57.0	wwPDB-VP

Xtriage's analysis on translational NCS is as follows: *The largest off-origin peak in the Patterson function is 4.20% of the height of the origin peak. No significant pseudotranslation is detected.*

[1] Intensities estimated from amplitudes.

5 Model quality (i)

5.1 Standard geometry (i)

The Z score for a bond length (or angle) is the number of standard deviations the observed value is removed from the expected value. A bond length (or angle) with $|Z| > 5$ is considered an outlier worth inspection. RMSZ is the root-mean-square of all Z scores of the bond lengths (or angles).

Mol	Chain	Bond lengths		Bond angles	
		RMSZ	#$\|Z\|$ >5	RMSZ	#$\|Z\|$ >5
1	D	0.21	0/662	0.47	0/914
2	A	0.24	0/1884	0.46	0/2542
2	C	0.24	0/1884	0.49	1/2542 (0.0%)
3	B	0.22	0/682	0.47	0/940
All	All	0.23	0/5112	0.47	1/6938 (0.0%)

There are no bond length outliers.

All (1) bond angle outliers are listed below:

Mol	Chain	Res	Type	Atoms	Z	Observed(^{o})	Ideal(^{o})
2	C	200	LEU	CA-CB-CG	5.69	128.39	115.30

There are no chirality outliers.

There are no planarity outliers.

5.2 Close contacts (i)

In the following table, the Non-H and H(model) columns list the number of non-hydrogen atoms and hydrogen atoms in the chain respectively. The H(added) column lists the number of hydrogens added by MolProbity. The Clashes column lists the number of clashes within the asymmetric unit, and the number in parentheses is this value normalized per 1000 atoms of the molecule in the chain. The Symm-Clashes column gives symmetry related clashes, in the same way as for the Clashes column.

Mol	Chain	Non-H	H(model)	H(added)	Clashes	Symm-Clashes
1	D	645	0	678	6	0
2	A	1846	0	1898	17	0
2	C	1846	0	1898	11	0
3	B	665	0	697	7	0
4	S	163	0	0	1	0
All	All	5165	0	5171	37	0

Clashscore is defined as the number of clashes calculated for the entry per 1000 atoms (including hydrogens) of the entry. The overall clashscore for this entry is 4.

The worst 5 of 37 close contacts within the same asymmetric unit are listed below.

Atom-1	Atom-2	Distance(Å)	Clash(Å)
3:B:19:LEU:HD23	3:B:20:PRO:HD2	1.76	0.67
2:A:122:ASP:OD2	2:A:127:GLY:N	2.30	0.63
2:A:123:ALA:O	2:A:124:ASN:HB2	2.00	0.61
1:D:19:LEU:HD23	1:D:20:PRO:HD2	1.83	0.61
1:D:19:LEU:HD13	2:C:222:ARG:HG2	1.86	0.56

There are no symmetry-related clashes.

5.3 Torsion angles

5.3.1 Protein backbone ⓘ

In the following table, the Percentiles column shows the percent Ramachandran outliers of the chain as a percentile score with respect to all X-ray entries followed by that with respect to entries of similar resolution.

The Analysed column shows the number of residues for which the backbone conformation was analysed, and the total number of residues.

Mol	Chain	Analysed	Favoured	Allowed	Outliers	Percentiles	
1	D	84/86 (98%)	82 (98%)	2 (2%)	0	100	100
2	A	234/236 (99%)	230 (98%)	3 (1%)	1 (0%)	43	50
2	C	234/236 (99%)	228 (97%)	6 (3%)	0	100	100
3	B	86/88 (98%)	85 (99%)	1 (1%)	0	100	100
All	All	638/646 (99%)	625 (98%)	12 (2%)	1 (0%)	56	67

All (1) Ramachandran outliers are listed below:

Mol	Chain	Res	Type
2	A	124	ASN

5.3.2 Protein sidechains ⓘ

In the following table, the Percentiles column shows the percent sidechain outliers of the chain as a percentile score with respect to all X-ray entries followed by that with respect to entries of similar resolution. The Analysed column shows the number of residues for which the sidechain conformation was analysed, and the total number of residues.

Mol	Chain	Analysed	Rotameric	Outliers	Percentiles	
1	D	78/78 (100%)	78 (100%)	0	100	100
2	A	207/207 (100%)	207 (100%)	0	100	100
2	C	207/207 (100%)	207 (100%)	0	100	100
3	B	80/80 (100%)	80 (100%)	0	100	100
All	All	572/572 (100%)	572 (100%)	0	100	100

There are no protein residues with a non-rotameric sidechain to report.

Some sidechains can be flipped to improve hydrogen bonding and reduce clashes. All (1) such sidechains are listed below:

Mol	Chain	Res	Type
2	C	22	GLN

5.3.3 RNA (i)

There are no RNA chains in this entry.

5.4 Non-standard residues in protein, DNA, RNA chains (i)

There are no non-standard protein/DNA/RNA residues in this entry.

5.5 Carbohydrates (i)

There are no carbohydrates in this entry.

5.6 Ligand geometry (i)

There are no ligands in this entry.

5.7 Other polymers (i)

There are no such residues in this entry.

5.8 Polymer linkage issues

There are no chain breaks in this entry.

6 Fit of model and data (i)

6.1 Protein, DNA and RNA chains (i)

In the following table, the column labelled '#RSRZ> 2' contains the number (and percentage) of RSRZ outliers, followed by percent RSRZ outliers for the chain as percentile scores relative to all X-ray entries and entries of similar resolution. The OWAB column contains the minimum, median, 95th percentile and maximum values of the occupancy-weighted average B-factor per residue. The column labelled 'Q< 0.9' lists the number of (and percentage) of residues with an average occupancy less than 0.9.

Mol	Chain	Analysed	<RSRZ>	#RSRZ>2			OWAB(Å^2)	Q<0.9
1	D	86/86 (100%)	0.49	6 (6%)	16	24	36, 49, 85, 124	0
2	A	236/236 (100%)	0.45	11 (4%)	30	41	37, 55, 94, 116	0
2	C	236/236 (100%)	0.42	8 (3%)	43	54	34, 51, 93, 115	0
3	B	88/88 (100%)	0.44	6 (6%)	17	25	42, 55, 89, 116	0
All	All	646/646 (100%)	0.44	31 (4%)	29	41	34, 53, 93, 124	0

The worst 5 of 31 RSRZ outliers are listed below:

Mol	Chain	Res	Type	RSRZ
1	D	103	THR	6.7
1	D	19	LEU	6.6
3	B	103	THR	5.9
3	B	18	ARG	4.9
1	D	104	PRO	4.8

6.2 Non-standard residues in protein, DNA, RNA chains (i)

There are no non-standard protein/DNA/RNA residues in this entry.

6.3 Carbohydrates (i)

There are no carbohydrates in this entry.

6.4 Ligands (i)

There are no ligands in this entry.

6.5 Other polymers (i)

There are no such residues in this entry.
